# Elevation of brain glucose and polyol-pathway intermediates with accompanying brain-copper deficiency in patients with Alzheimer’s disease: metabolic basis for dementia

**DOI:** 10.1038/srep27524

**Published:** 2016-06-09

**Authors:** Jingshu Xu, Paul Begley, Stephanie J. Church, Stefano Patassini, Selina McHarg, Nina Kureishy, Katherine A. Hollywood, Henry J. Waldvogel, Hong Liu, Shaoping Zhang, Wanchang Lin, Karl Herholz, Clinton Turner, Beth J. Synek, Maurice A. Curtis, Jack Rivers-Auty, Catherine B. Lawrence, Katherine A. B. Kellett, Nigel M. Hooper, Emma R. L. C. Vardy, Donghai Wu, Richard D. Unwin, Richard L. M. Faull, Andrew W. Dowsey, Garth J. S. Cooper

**Affiliations:** 1 School of Biological Sciences, and Maurice Wilkins Centre for Molecular Biodiscovery, Faculty of Science, University of Auckland, New Zealand; 2 Centre for Brain Research, Faculty of Medical and Health Sciences, University of Auckland, New Zealand; 3 Centre for Advanced Discovery and Experimental Therapeutics (CADET), Central Manchester University Hospitals NHS Foundation Trust, and Institute of Human Development, Faculty of Medical and Human Sciences, University of Manchester, United Kingdom; 4 Institute of Brain, Behaviour and Mental Health, Faculty of Medical and Human Sciences, University of Manchester, United Kingdom; 5 Anatomical Pathology, LabPLUS, Auckland City Hospital, Auckland, New Zealand; 6 Salford Royal NHS Foundation Trust, Salford, United Kingdom; 7 Guangzhou Institutes of Biomedicine and Health, Chinese Academy of Sciences, Guangzhou, China

## Abstract

Impairment of brain-glucose uptake and brain-copper regulation occurs in Alzheimer’s disease (AD). Here we sought to further elucidate the processes that cause neurodegeneration in AD by measuring levels of metabolites and metals in brain regions that undergo different degrees of damage. We employed mass spectrometry (MS) to measure metabolites and metals in seven *post-mortem* brain regions of nine AD patients and nine controls, and plasma-glucose and plasma-copper levels in an *ante-mortem* case-control study. Glucose, sorbitol and fructose were markedly elevated in all AD brain regions, whereas copper was correspondingly deficient throughout (all *P* < 0.0001). In the *ante-mortem* case-control study, by contrast, plasma-glucose and plasma-copper levels did not differ between patients and controls. There were pervasive defects in regulation of glucose and copper in AD brain but no evidence for corresponding systemic abnormalities in plasma. Elevation of brain glucose and deficient brain copper potentially contribute to the pathogenesis of neurodegeneration in AD.

“Alzheimer’s disease” (AD) is the predominant cause of ageing-related dementia^1^ and refers to a constellation of cognitive and behavioural changes that are typical for patients who have substantial amounts of its hallmark pathological lesions in their brain^2^. AD may occur through interplay of environmental, genetic, and metabolic factors^3^,^4^, but some brain regions are impacted more severely than others, consistent with operation of a specific disease mechanism^5^,^6^. The pathophysiological process of AD is thought to begin many years before the diagnosis of AD-related dementia^7^,^8^, and sporadic AD accounts for the vast majority of cases^2^. AD is a non-communicable disease which displays several pathogenic mechanisms in brain, including: Aβ-amyloid deposition^9^; neurofibrillary tangles comprising tau protein^2^,^8^; impaired cerebral glucose metabolism (‘hypometabolism’) with insulin resistance and defective carbohydrate regulation^8^,^10^; oxidative stress and inflammation^4^,^11^; cerebrovascular amyloid angiopathy (CAA)^12^; enhanced advanced glycation end-product (AGE) formation^9^; and defective copper regulation^13^,^14^. Risk of sporadic AD has also been linked to presence of type-2 diabetes (T2D) in epidemiological studies^15^,^16^.

Glucose uptake^17^,^18^ and regional cerebral perfusion^6^ are impaired in AD, particularly in severely-damaged brain regions^5^,^19^, and progressive reductions in glucose-uptake may occur years before clinically-evident dementia^19^: however, the molecular basis for these defects is uncertain. Impaired brain-glucose regulation has been tentatively attributed to a functional defect of glucose transporter (GLUT)-mediated glucose uptake^20^, but neuronal loss may be responsible for some of this effect, and there is ongoing argument concerning the contribution of this mechanism to defective brain-glucose uptake and neurodegeneration.

Here we analyzed *post-mortem* tissue from seven brain regions of patients with AD and controls in whom clinical dementia had been excluded and who had no *ante-mortem* evidence for neurodegeneration or T2D. Diabetes was excluded in this *post-mortem* study on the basis of a negative history and lack of any biochemical evidence of hyperglycemia but not by systematic *ante-mortem* glucose-tolerance testing: it is therefore possible, albeit unlikely, that one or more patients had undiagnosed mild T2D or prediabetes. Elevated glucose levels have previously been linked to altered tissue-copper regulation in the context of diabetes^21^. Furthermore, in AD, some brain regions are more severely damaged than others^22^,^23^. Therefore, we measured tissue levels of metabolites and trace metals^24^,^25^ in seven human brain-regions in patients and controls matched for age and gender: three of these undergo severe neuronal damage in AD (hippocampus, entorhinal cortex and middle temporal gyrus); three are less affected (cingulate gyrus, sensory cortex and motor cortex); and one (cerebellum) is relatively spared^5^,^6^,^22^. We also measured levels of brain metabolites and copper in (i) *post-mortem* whole-brain tissue of diabetic and control rats, to probe potential effects of *post-mortem* delay (PMD) on metabolite levels and contrast effects of AD with those of diabetes in an animal model; and (ii) metabolites and metal levels in brains from a triple-transgenic mutant model of familial AD (fAD) and controls^26^. Finally, we measured fasting-plasma glucose (FPG), hemoglobin A_1c_ (HbA1c), and fasting-plasma copper in AD-patients and controls from a clinical case-control study, to ascertain whether patients might show evidence of elevated systemic glucose consistent with diabetes or lesser degrees of hyperglycemia, or signs of impaired systemic copper metabolism.

## Results

### Post-mortem study of AD brain: group characteristics

All participants in this study were from New Zealand and were matched for age, gender and PMD (Tables 1 and 2). Median (range) brain-weight was 1062 (831–1355) g in cases and 1260 (1094–1461; *P* = 0.005) in controls (Tables 1 and 2): the ~16% decline in brain-weight in AD is consistent with histological severity^22^. The case-control matching and short PMD support the quality of these data (Tables 1 and 2). Cases were diagnosed with AD by clinical^27^, CERAD^28^ and Braak criteria^22^: patients had late-onset/sporadic AD and none had findings consistent with early-onset AD, fAD, or T2D.

### Glucose levels in AD brain

**Figure 1:**
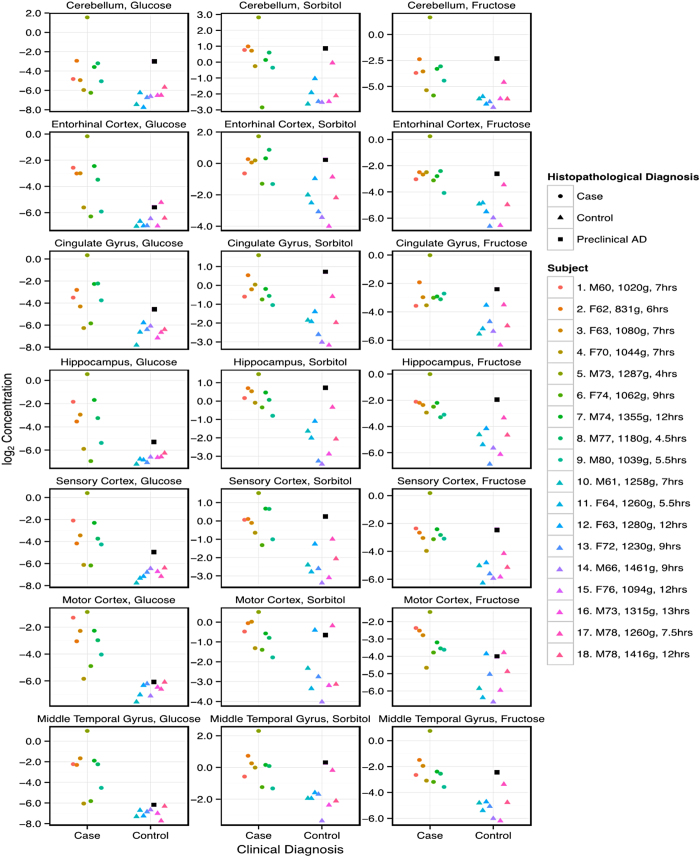
Log_2_-transformed concentrations of glucose, sorbitol and fructose in *post-mortem* tissue from seven brain regions of nine patients with AD and nine controls. Individual values are shown for each region. At the right-hand side are detailed the identity, sex, age, brain-weight, and PMD of each participant, along with the histopathological diagnosis. One control (Case 15: F76, brain wt 1094 g; *black squares*) with higher metabolite concentrations than other controls had preclinical AD (Braak Stage II).

**Figure 2:**
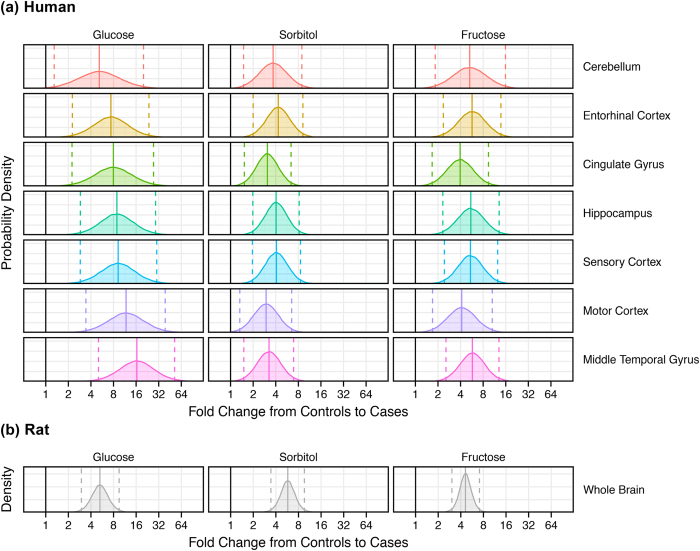
Probability density distributions of fold-changes from controls to cases in glucose, sorbitol and fructose measured in brain tissue from (**a**) each of seven brain regions from patients with AD (n = 9) and controls (n = 9), and **(b)** whole brain homogenates from diabetic (n = 7) and control (n = 7) rats. Distributions were derived by Bayesian modeling and illustrate the plausible ranges (posterior distributions) of inferred fold changes for each region and metabolite. This analysis complements the results of a mixed-effects model assessment of the fold-change between AD cases and controls for measurements of metabolites in each brain region. Posterior means (*solid lines*) and 95% credible intervals (*dashed lines*) are shown, along with the ‘no fold change’ value (*solid black line*). **(b)** Shown are results of an equivalent simulation of data from a metabolomic study of *ex-vivo* whole-brain tissue from diabetic rats and controls. Data were log_2_-transformed before analysis.

**Figure 3:**
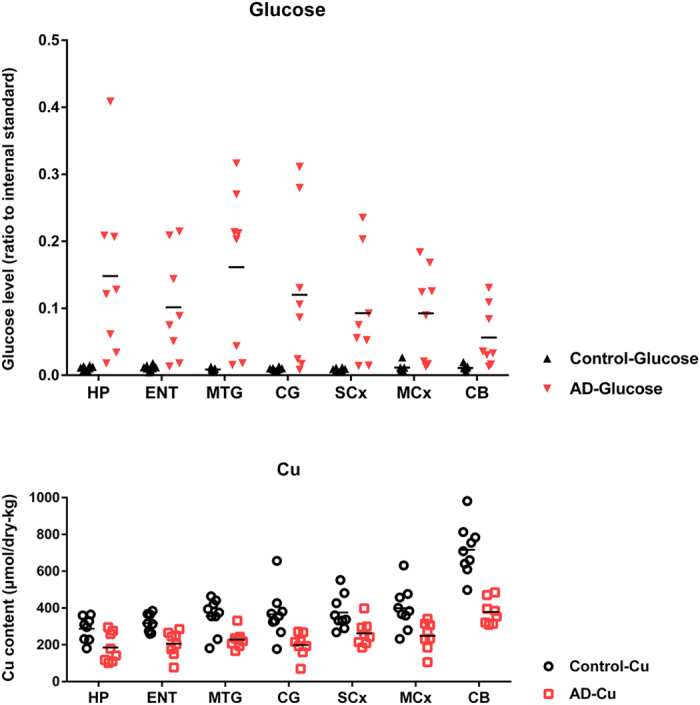
Regional brain glucose (*upper panel*) and brain copper (μmol/dry kg; *lower panel*) levels in patients with AD (n = 7–9/region; *red*) and controls (n = 7–9; *black*). Overall mean ( ± 95% CI; Confidence Interval) wet-weight/dry-weight ratio was 5.6 (5.4–5.8). Overall mean brain-glucose levels were higher (*P* = 9.7 × 10^−13^) and brain copper values lower (*P* = 1.6 × 10^−8^) in cases than controls. Mean brain copper in AD trended lower in every region: CB (*P* < 0.0001); MCx (*P* = 0.021); SCx (*P* = 0.027); CG (*P* = 0.0079); MTG (*P* = 0.013); ENT (*P* = 0.0046); and HP (*P* = 0.070); and there was modest evidence that overall brain-copper and brain-glucose values were inversely correlated (*P* = 0.021) in AD-patients but not controls. Abbreviations: CB, cerebellum; CG, cingulate gyrus; ENT, entorhinal cortex; HP, hippocampus; MCx, motor cortex; MTG, middle temporal gyrus; SCx, sensory cortex.

Levels of glucose were elevated in all brain regions of AD-patients (Table 3, Figs 1, 2, 3). Mean (±95% CI) elevations in glucose varied from 5.2-fold (1.4–18.6) in cerebellum to 16.4-fold (5.2–51.2) in middle temporal gyrus (Figs 1 and 2a). There was evidence for differences in glucose elevations between different brain regions in AD where levels tended to be higher in more severely affected regions such as the middle temporal gyrus (Table 3; Figs 1 and 2a). Elevated glucose levels can damage proteins and lipids through chemical attack via the aldehyde group, forming AGEs in diabetic tissues^9^,^29^. Our data indicate that potential exists for similar glucose-driven damage mechanisms to occur in AD brain, consistent with co-localization there of AGEs with Aβ-containing plaque^9^. Variation in PMD did not affect glucose values here (data not shown).

### Sorbitol levels in AD brain

Sorbitol, formed from glucose, is the first metabolite in the polyol pathway, which usually accounts for a few percent at most of glucose utilization under normal conditions^30^. Here, sorbitol was elevated in all AD brain regions: mean (±95% CI) fold-elevations ranged from 3.0 (1.4–6.6) in motor cortex to 4.3 (2.0–9.1) in entorhinal cortex (Figs 1 and 2; Table 3). Variation in sorbitol levels was less than for glucose and there was no evidence for inter-regional differences, possibly reflecting sorbitol formation from glucose as the rate-limiting step in the polyol pathway. Variation in PMD did not affect sorbitol levels.

### Fructose levels in AD brain

Fructose is the second metabolite in the polyol pathway^30^. Fructose may be even more reactive in AGE-mediated damage mechanisms than glucose, and levels of the two are similar in AD brain. Here, brain-fructose levels were elevated in all cases, consistent with the findings for glucose and sorbitol (Figs 1 and 2a; Table 3). Elevations in brain fructose showed less variation than glucose, consistent with sorbitol: fold-elevations were between 3.9 (1.7–9.2) in cingulate gyrus and 5.7 (2.6–12.9) in middle temporal gyrus. Inter-regional differences were not significant and variation in PMD did not affect fructose levels.

### Inter-regional comparisons of metabolite levels in human brain

Mean elevations in glucose fold-change, 9.5 (3.0–30.4) were significantly higher across all regions in AD brain than those for sorbitol and fructose (*P* = 0.0002). Values for sorbitol, 3.6 (1.7–7.8), and fructose, 5.1 (2.1–12.2) did not differ significantly across regions. Mean values of all three metabolites were substantively elevated in all AD brain regions (Figs 1 and 2): these elevations were widespread in AD, consistent with a global defect.

### Post-mortem brain metabolites in a patient with preclinical AD

Elevated brain levels of glucose, sorbitol and fructose were present in one control patient, a 76 year-old female (Code H241) who had no *ante-mortem* clinical evidence for brain disease or dementia, but had preclinical AD characterized by low brain-weight (1,094 g) and positive *post-mortem* histology (Braak Stage II; ABC score^2^ of A3, B1, C1; *black squares* in Fig. 1) consistent with “AD neuropathologic change”^2^. This finding indicates that substantive disturbance of brain glucose and polyol pathway regulation may antedate the onset of clinical dementia, possibly by a considerable period.

### Copper levels in AD brain

Mean (±95% CI) brain-copper levels summed across all seven regions were 256 (232–279) μmol/dry kg in AD and 406 (363–449) μmol/dry kg in controls (*P* = 1.6 × 10^−8^): there was thus an overall decrease of ~40% in brain-copper levels in AD. Mean brain-copper levels tended to be lower in AD than controls in all brain regions examined (Fig. 3). In addition, there was moderate evidence for a trend in copper levels to be inversely proportional to tissue-glucose values in AD-patients (*P* = 0.021; restricted iterative generalized least squares^31^) but not in controls.

### Levels of tissue metabolites and copper in rodent brain

We did not have access to *post-mortem* brain from diabetic patients with dementia suitable for this study. Instead, we measured levels of metabolites and trace metals in whole brains from male rats with streptozotocin-induced diabetes (a model of severe T1D)^21^ and whole mouse brains from a model of AD (13–14-month-old male triple-transgenic mice)^26^, by applying the same methods we used for analysis of human brain. Diabetic-rat brain displayed marked elevations in glucose, sorbitol, and fructose, similar to those in human AD brain (Suppl. Table S1; Fig. 2b; all *P* < 0.0001). Thus, elevations in *post-mortem* human AD brain tissue were similar to those in rat-brain snap-frozen within ~1 minute after death. By contrast, there was no significant difference in brain-copper levels between diabetic and control rats: mean (±95% CI) copper values were 209.0 (189.8–228.1) μmol/dry-kg in diabetic rats and 188.4 (147.1–229.8) μmol/dry-kg in controls.

In the triple-transgenic model of AD^26^, brain-glucose levels were below the limits of detection (~1–5 μmol/kg) in both experimental groups; brain-fructose levels were lower in transgenic mice than controls (*P* = 0.0005); sorbitol levels trended lower in transgenic mice but differences were not significant; and copper values did not differ between transgenic mice and controls (data not shown).

### Case-control study of plasma glucose and plasma-copper levels in patients with AD

Participants in this case-control study were matched for gender and age (see Suppl. Table S2). Consistent with expectation, measured cognitive function scores (MMSE) in the AD group were significantly lower than those of controls whereas the ApoE4 allele was more prevalent. FPG and serum HbA1c levels were equivalent between groups so there was no evidence for elevated rates of undiagnosed T2D, impaired glucose tolerance (IGT) or impaired fasting glucose (IFG) in these British patients with late-onset AD. Fasting plasma-copper levels did not differ significantly between the AD group and controls (see Suppl. Table S2).

## Discussion

Glucose is the obligatory energy-generating substrate in the brain^32^, and impaired brain-glucose uptake and utilization is a key metabolic defect in AD^33^: this phenomenon is generally worse in brain regions with greater histological^3^,^34^, functional^19^ and molecular^4^ evidence of damage, consistent with the operation of a specific disease process^17^,^19^. Transport of glucose across the plasma membrane of mammalian cells is the first rate-limiting step for glucose metabolism and is mediated by facilitative GLUT proteins^35^. One potential explanation for decreased brain-glucose uptake could be that of impaired GLUT activity, leading to consequent cerebral ‘hypometabolism’. Consistently, lowered levels of GLUT1, the main glucose transporter in the blood-brain barrier (BBB), have been reported in several studies of AD^20^,^36^,^37^,^38^,^39^; many GLUT-related changes in AD pathogenesis may occur before the onset of neuronal dysfunction^40^. A reduction of GLUTs at the BBB that occurs before the onset of the main pathophysiological changes and symptoms of AD could contribute to pathogenesis^39^. However, direct measurements of glucose concentrations in AD brain tissue have hitherto been lacking.

Unexpectedly, we found here that glucose levels in AD were markedly elevated rather than decreased in all seven brain regions of these patients with late-onset AD: therefore, diminished cerebral GLUT activity is unlikely to play an initiating role in decreased glucose uptake in AD. Rather, these data indicate that lower brain-GLUT/glucose-uptake rates in AD probably occur *in response to* elevated brain-glucose levels; consistently, there is substantive evidence that elevated cellular glucose can mediate down-regulation of GLUT1 expression and function^41^,^42^. Elevated brain-glucose levels may therefore antedate the occurrence of GLUT down-regulation at the BBB in the pathogenesis of AD. The presence of elevated brain-glucose levels in AD accords with impaired intracellular glucose utilization through glycolysis and the tricarboxylic acid (TCA) cycle: consistently, there is substantive evidence for defects in AD of TCA-related mitochondrial enzymes including the pyruvate dehydrogenase complex, the α-ketoglutarate dehydrogenase complex, and cytochrome c oxidase/complex IV^43^.

Here, marked elevations in levels of sorbitol and fructose were also present in all AD brain regions. These metabolites are produced from glucose via the polyol pathway in neurons and astrocytes^44^. The proportion of cortical oxidative metabolism attributable to astrocytes, ~30%, roughly corresponds to their volume fraction, indicating that astrocytes and neurons have similar fuel oxidation rates^45^, so both cell-types could contribute to this effect, in proportions depending on the relative distribution of polyol-pathway intermediates in the two.

Brain-glucose levels were lower in cerebellum than other regions whereas elevations in sorbitol and fructose were similar in all regions studied (Fig. 2). Glucose levels trended higher in regions more prone to tissue-damage in AD, consistent with insufficient clearance via glycolysis with consequent up-regulation of the alternative polyol pathway^29^. The human brain comprises similar numbers of neurons and non-neuronal cells^46^: both make substantive contributions to functional *in vivo* indices of energy metabolism^47^, although non-neuronal cells have more prominent gluconeogenesis and glycogenesis^48^,^49^. It will therefore be important to determine whether the elevation of glucose and polyol-pathway intermediates in AD is caused by metabolic defects in neurons, glia, or both.

Our measurements of FPG and HbA1c^50^ in this group of patients with AD provide no evidence for increased prevalence of undiagnosed T2D, IGT or IFG (see Suppl. Table S2): therefore, it is unlikely that dementia in these patients occurred as a result of systemic disorders of glucose metabolism such as diabetes or IGT. Rather, our findings indicate that defective glucose metabolism in AD may occur only in a *restricted location*, the brain. Our data in brain tissue from AD patients and rats with severe diabetes were similar with respect to their glucose, sorbitol and fructose content. Therefore, the brain in patients with AD presents an abnormality of glucose utilization closely resembling that in the brain (the current study) and peripheral nervous tissue (sciatic nerve, dorsal root ganglia, and trigeminal ganglia) of the rats with severe diabetes^51^.

Our finding of elevated blood glucose, sorbitol and fructose in a patient with Braak Stage-II disease without clinical evidence of dementia is potentially significant, and suggests that metabolic disturbances in AD- brain may occur relatively early in disease progression. It was recently reported that acute hyperglycemia in young AD-model mice had increased Aβ production in the interstitial fluid, which was augmented in aged AD-mice with marked Aβ plaque pathology, identifying a mechanism by which systemic glucose elevation could damage brain cells^52^. However, in light of our current findings of marked elevations of brain glucose, sorbitol and fructose levels in AD *without* systemic elevation of glucose, direct toxicity caused by isolated *intra-cerebral* metabolite elevations could provide an alternative mechanism for neurodegeneration in AD.

How might elevated brain levels of glucose, sorbitol and fructose cause neurodegeneration? One potential mechanism is that glucose (an aldehyde) and fructose (a ketone) can attack macromolecules through their reactive functional groups leading to AGE formation, as occurs in the diabetic complications^29^. Tissues in AD brain are known to be modified by N-epsilon-carboxymethyllysine (CML) formation^9^,^53^, which coordinates divalent copper^9^,^13^,^54^, localizing it to affected regions and thereby enhancing localized pro-oxidant stress^55^,^56^; consistent with this mechanism, copper homeostasis is evidently impaired in AD (summarized in)^13^. These mechanisms may well make similar contributions to AGE formation and downstream pathogenesis in AD.

Brain glucose uptake in AD is lower in severely affected regions and occurs early in disease development^18^. For example, using 2-[^18^F]fluoro-2-deoxy-D-glucose positron-emission tomography (FDG-PET), it has been shown that there is diminished glucose uptake in the hippocampus, parietotemporal cortex and/or posterior cingulate cortex in: (i) individuals who are at genetic risk for fAD^57^,^58^; (ii) individuals with a history of AD^59^; as well as (iii) those who have mild or no cognitive impairment but eventually go on to develop AD^19^. The relevance of studies in fAD to those of sporadic AD are uncertain; there is thus an identified need for similar metabolomic and metallomic studies in suitable cohorts of fAD patients. Elevated intracellular glucose diminishes the trans-membrane gradient that drives facilitative glucose uptake via glucose transporters, so these findings in fAD are not inconsistent with the current results: however, levels of brain glucose and polyol pathway intermediates in fAD are currently unknown. The findings in (iii) indicate that impaired cerebral glucose uptake presages the development of (presumably sporadic) AD: these findings are consistent with elevated brain glucose as a key early aspect of pathogenesis.

It has also been shown that, before neuronal dysfunction develops, mice heterozygous for GLUT1 (Slc2a1) at the BBB develop cerebral blood flow perfusion deficits and BBB breakdown that leads to vascular-mediated neurodegeneration^40^: the relevance of this study in engineered mice to our findings in human brain is uncertain, given that these authors did not report brain-glucose levels. Our own findings show that adult triple-mutant mice did not develop elevated brain glucose, and that patterns of sorbitol and fructose levels differed markedly from those in patients with AD; we therefore conclude that these mice may not reflect disturbances of glucose and the polyol pathway that occur in humans with Alzheimer’s dementia, although the proviso remains that the disease in these mice is highly region-specific and may not have generated the widespread dysregulation present in patients. Further studies in this and other transgenic models of AD may be indicated. Here we employed male transgenic mice, to avoid the increased metabolic variability which can occur in female mice and is attributable to effects of the estrus cycle, and in light of the similar prevalence of AD in male and female patients. Study of only male transgenic mice may have affected our results since Dr LaFerla, who developed this model, has recently reported that male triple-transgenic mice may not exhibit the phenotypic traits originally described (see: https://www.jax.org/strain/004807). Prevalence of AD is similar in male and female patients, so the potential sexual dimorphism in this model may represent another difference between it and the human disease process. Further studies in this and other transgenic models of AD are therefore indicated in order to ascertain whether any of the available models might demonstrate defective glucose and polyol pathway regulation similar to that present in AD brain.

In glycolysis, hexokinase and phosphofructokinase catalyse reactions where ATP is the phosphate-group donor. Current results could be consistent with inefficient glucose utilization caused by impaired glycolysis and consequent elevation in polyol-pathway metabolites; consistent with this interpretation, for example, hippocampal lactate was elevated by 2.6-fold in cases compared with controls; fold-values (case-control ratios) measured in other brain regions were as follows: EC, 0.5; MTG, 1.3; SCx, 3.4; MCx, 7.3; CG, 1.7; CB, 0.6; thus values in SCx and MCx showed apparent upward trends, although these were not significant, possibly due to the limited numbers in each group. Further study of lactate levels in larger numbers of cases and controls will be required to confirm and extend these data. Elevation of brain glucose and polyol-pathway intermediates might be explained by impaired ATP supply leading to diminished glycolysis, suppressing these ATP-requiring phosphorylation reactions, and could ultimately result from impaired TCA cycle function^60^. Additionally, in the physiological cerebral *milieu*, glycogen provides a metabolically-active storage form of glucose in astrocytes^61^,^62^. Therefore, it is possible that some of the elevated free glucose we measured in AD brains could have been derived from increased rates of glycogen breakdown via glycogenolysis; alternatively, elevated glucose could mediate increased deposition of brain glycogen or other forms of polymerized glucose^63^ such as metabolically-inactive, high-molecular-weight amylose that may be formed at the expense of glycogen in AD brains, thereby decreasing the pool of metabolically-accessible stored glucose^63^.

Elevated free glucose promotes glycation of macromolecules leading to formation of CML. Here, our finding of elevated brain glucose in AD provides a molecular explanation for previously-reported elevations of CML in AD brain^9^. PET-imaging studies in AD, where the rate-constant k_2_ is ~normal^64^, argue against substantively-elevated cytoplasmic free glucose available for retrograde transport into the extracellular space. PET findings are not inconsistent with the current findings, however, with respect to large elevations in tissue-glucose since it binds to macromolecules in a form releasable upon chemical extraction. For example, formation of Schiff-base (aldimine) linkages to proteins is the first step in a reversible pathway leading to CML formation^65^, reconciling published imaging results with elevated brain-glucose levels; additionally, fructose binds to proteins through an equivalent mechanism^66^.

Cells require a substantial, uninterrupted copper supply to maintain effective levels of metabolic-fuel utilization and anti-oxidant defense^67^. Copper for support of fuel utilization is required particularly by the copper-enzymes cytochrome oxidase I (COI) and COII (both inner-membrane components of mitochondrial respiratory-chain complex IV), and for anti-oxidant defense by copper-enzymes superoxide dismutase 1 (SOD1, intracellular) and SOD3 (extracellular)^21^,^68^. Deficient enzyme-bound copper caused by defective cell-copper uptake impairs enzyme function that can cause metabolic sequelae including severe tissue damage^21^,^69^. The brain has particularly large copper requirements compared with most other organs, since it is very metabolically active^70^ and generates large amounts of reactive oxygen species, particularly superoxide anion that requires clearance by SOD1 and SOD3. In this context, our current findings of severely-elevated glucose coupled with markedly-deficient copper levels in AD brain are significant, particularly given that elevated CML levels are also widespread in AD brain^9^. CML directly links elevated tissue-glucose with low tissue-copper, since increased tissue-glucose levels drive CML-modification of collagen^71^, which inhibits cell-copper uptake by suppressing cell-membrane copper transport via copper transporter 1^21^. A similar process occurs in diabetic heart, where cardiac copper is deficient along with hyperglycemia and elevated coronary CML^21^, causing myocardial-copper deficiency that mirrors the current findings in AD brain and causes defective copper supply to SOD1 and cytochrome c oxidase/complex IV^13^,^21^. There is substantive evidence that defective tissue-copper uptake is triggered by coordination of bioactive divalent copper to CML in collagen^54^. These findings provide a new and potentially reversible mechanism for the causation of brain damage in AD. Whether this mechanism can be targeted by pharmacological intervention in AD remains to be determined.

This study has limitations. There is evidence that brain from significant numbers of aged but cognitively unimpaired patients may display elevated Braak stages, not infrequently up to III-IV^72^,^73^, whereas by contrast, our group of 9 controls showed scores of 0-II. In several studies, Braak scores in the range of 0-II have been reported to occur in more than half of all cognitively-normal elderly persons^73^,^74^,^75^, so our sample may well be consistent with derivation from a similar population. Knopman *et al*. concluded that “the majority of individuals who are cognitively normal near the time of their death have minimal amounts of tau-positive neuritic pathology (that is, Braak stage <IV)”^73^. Cases at the more severe end of the pathologic spectrum (Braak stages III-IV) lacking Aβ plaques have been reportedly reported in 2–10% of brains in large autopsy series that included community-based sampling^75^,^76^,^77^. Thus it is not entirely clear whether our control group is representative of the status of aged patients without dementia: larger follow-up studies will be required in clinic-based and community-based cohorts to avoid selection bias^77^ and thereby to better characterise the status of the polyol pathway in aged patients with and without varying degrees of cognitive impairment.

Despite the limited numbers (n = 9 cases and n = 9 controls), our findings in seven brain regions are statistically robust; however, they require replication in other cohorts of AD patients, and also extension to subjects with preclinical AD, mild cognitive impairment (MCI), and fAD syndromes. Data from the single patient with preclinical AD reported here provide compelling, albeit preliminary data that similar metabolic perturbations might occur in other patients with preclinical disease, but this remains to be verified in suitable larger studies. Equivalent post-mortem metabolomic studies in preclinical AD, MCI, and fAD will require studies of suitable brain tissue from cohorts of patients with these conditions, which are likely to be challenging, and lie beyond the scope of the current study. We did not have the opportunity of performing *ante-mortem* assessments by MMSE or CDR scoring in the *post-mortem* study: this represents a limitation that will only be addressed once suitable brain tissue from *post-mortem* cohorts of patients who have previously been characterized by MMSE/CDR scoring are analyzed by suitable metabolomic methods. The current study does, however, open the field up to consideration of the roles played by elevations in brain glucose and polyol-pathway metabolites and associated copper deficiency in the pathogenesis of AD.

In conclusion, we have shown here that free glucose is markedly elevated in AD brain, particularly in vulnerable regions where elevated glucose may well be linked to copper deficiency and tissue-damage. Brain-sorbitol and brain-fructose levels are also elevated in AD brain, consistent with increased polyol-pathway flux, potentially caused by defective glucose utilization via glycolysis and/or the TCA-cycle. A further, mechanistically-related finding is that pan-cerebral brain-copper deficiency accompanies these glucose and polyol-pathway defects: elevated tissue-CML levels provide a molecular linkage between elevated brain glucose and copper deficiency. Impaired neuronal glucose utilization could play a major role in tissue-damage in AD, possibly via defective mitochondrial metabolism caused by deficient copper supply to COI and COII, leading to impaired function of cytochrome c oxidase^78^. These findings provide a clear molecular linkage between mechanisms of tissue damage in AD and T2D. The process by which elevated brain glucose is linked to defective cell-copper uptake and intracellular transport to key copper proteins such as COI, COII, SOD1 and SOD3, may well provide a new target for diagnostic imaging or therapeutic intervention.

## Methods

### Ethics

All experiments were performed in accordance with relevant guidelines and regulations as stated below: the case-control study of *post-mortem* human brain was approved by the University of Auckland Human Participants Ethics Committee with informed consent from all families. The observational case-control study was performed under a protocol approved by the Leeds Teaching Hospitals NHS Trust Research Ethics Committee with informed consent from participants or families of patients.

The studies in the streptozotocin-induced rat model of diabetes and the triple-transgenic mouse model of AD were performed under UK Home Office Licenses, according to protocols approved by the University of Manchester Animal Welfare and Ethical Review Body (AWERB), and were consistent with the Animals (Scientific Procedures) Act 1986 and the ARRIVE guidelines.

### Human brains: acquisition and analysis

Human brains were obtained from the New Zealand Neurological Foundation Human Brain Bank, University of Auckland^79^. Tissue quality was confirmed by demonstration of mRNA integrity (data not shown). Each brain was dissected under the supervision of neuroanatomists (JX, SP, HJW and RLMF), who accurately identified each region. *Ante-mortem* MMSE and CDR (Clinical Dementia Rating) examinations were not performed on patients enrolled in the study of *post-mortem* human brain tissue. Tissue was analyzed for metabolites (~50–800 Da) and copper: regions studied were entorhinal cortex, hippocampus, middle temporal gyrus, cingulate gyrus, sensory cortex, motor cortex, and cerebellum: grey matter from each region was sampled as shown (Suppl. Fig. S1). Aliquots of 50 ± 5 mg were dissected from each region and stored at −80 °C until analysis, and were otherwise treated as previously described^4^. Patients had *ante-mortem* evidence of clinical dementia, whereas controls did not. Controls were selected by matching for age, sex and PMD (Tables 1 and 2). A consultant neuropathologist diagnosed or excluded AD by applying the Consortium to Establish a Registry for Alzheimer’s Disease (CERAD) criteria^28^, and determined the neuropathological severity by assigning the Braak stage^22^ and amyloid load by applying the 2013 consensus National Institute on Aging–Alzheimer’s Association guidelines^2^ (Tables 1 and 2).

### Data from one control participant with Braak Stage II disease

One control patient had neuropathological findings consistent with AD (Braak Stage II; Table 2) and was therefore diagnosed with preclinical disease: this finding is consistent with the known frequency of asymptomatic AD in similarly-aged groups in the study population^80^. To assess the impact of this patient’s data on the overall outcome, the main statistical analysis was performed twice, first by retaining the corresponding patient’s data in the control group (Table 3) and then by performing a second analysis using the same methods but excluding this patient’s data only: the two analyses yielded equivalent results so this patient’s data have been retained in the control group, despite the presence of some of the metabolic changes observed in diagnosed AD cases.

### Observational case-control study

Patients with memory disorder and control participants without cognitive impairment were recruited^81^ respectively through memory clinics in Leeds and Dewsbury (England), and the Leeds Family Health Services Authority day hospitals and elderly medicine outpatient clinics in the Leeds area. All were of European Caucasian background and gave written informed consent (consent from relatives of the AD-patients was provided, where appropriate). Diagnosis of probable AD was made in accordance with international diagnostic criteria (National Institute of Neurological and Communicative Disorders and Stroke-Alzheimer’s Disease and Related Disorders Association Work Group: NINCDS-ADRDA)^82^. All participants underwent a standardised clinical evaluation: medical history, fasting plasma glucose (FPG) and HbA1c, and cognitive function assessment by MMSE. AD samples were refined by excluding the few patients with diagnosed T1D or T2D, including those on insulin, or others with significant co-morbidities. Samples were then selected from the whole-study population for whom required measurements (FPG, HbA1c) were available^83^. The resulting 42 AD-patients were then age- and gender-matched to 43 corresponding controls.

### Analysis of rat brain

We employed a validated model of diabetes^21^ using 6-7 week-old male Wistar rats (n = 14), with body-weights of 220–250 g, maintained under a 12:12-h light:dark cycle and housed at 22 ± 2 °C and 60% humidity, with *ad libitum* access to standard rat chow (Special Diets Services, Dietex International, Essex, UK) and fresh water. Diabetes was induced (n = 7) by intraperitoneal injection of streptozotocin (55 mg/kg; Sigma Aldrich, Gillingham, UK) and diagnosed by blood-glucose levels of >15 mmol/l on two consecutive measurements^21^; non-diabetic control animals (n = 7) were age-matched littermates who received a single intraperitoneal injection of sodium-citrate buffer (vehicle) only. Eleven weeks after induction, we euthanized rats by terminal isoflurane anesthesia, excised brains, and stored them at −80 °C until analysis. Whole brains were homogenized and sampled to yield 50 ± 5 mg (wet-weight) aliquots. Equivalent methods were used for analysis of mouse-brain tissue.

### Transgenic mice: methods

Male triple transgenic (3xTgAD) mice expressing mutant PS1_M146V_, APP_Swe_, Tau_P301L_, and control non-transgenic (non-Tg, 129/C57BL6) mice, were originally supplied by Frank LaFerla (Irvine, CA, USA)^26^ and an in-house colony established at the University of Manchester. All mice were kept in standard housing conditions (humidity 50–60%, temperature 21 ± 1 °C, 12:12 hour light-dark cycle) and given *ad libitum* access to standard rodent chow (Special Diets Services). At 13–14 months of age, non-Tg (n = 10) and 3xTgAD (n = 10) were terminally anesthetized with 3-5% isoflurane (30% O_2_ and 70% N_2_O) and then transcardially perfused with 0.9% saline. The brains were quickly removed, and one hemisphere was snap-frozen on dry ice and stored at −80 °C until analysis.

### Analytical methods

More detailed descriptions are given in the Supplementary Materials. Briefly, metabolite levels were compared between cases and controls by gas chromatography mass spectrometry (GC-MS)-based metabolomics in wet-tissue^24^, copper was measured in dry-tissue by inductively-coupled-plasma mass spectrometry (ICP-MS)^25^, and statistical analysis was performed as described in the Supplementary Materials. *P*-values of < 0.05 were considered significant.

## Additional Information

**How to cite this article**: Xu, J. *et al*. Elevation of brain glucose and polyol-pathway intermediates with accompanying brain-copper deficiency in patients with Alzheimer’s disease: metabolic basis for dementia. *Sci. Rep.* **6**, 27524; doi: 10.1038/srep27524 (2016).

## Supplementary Material

Supplementary Information

## Figures and Tables

**Table 1:** Case-control study of *post-mortem* human brain in Alzheimer’s disease: group characteristics.

Variable	Control	Alzheimer’s disease
Number	9	9
Age	70.1 (6.7)	70.3 (7.1)
Male sex, n (%)	5 (55.6)	5 (55.6)
PMD (h)	9 (5.5–13.0)	7 (4.0–12.0)
Brain-weight (g)	1260 (1094–1461)	1062 (831–1355)*
Plaques, n (%)	1 (11)	9 (100)**
Tangles, n (%)	1 (11)	9 (100)**

Values are: age, mean (SD); PMD and brain-weights, median (range): *P = 0.005, **P < 0.0001 compared with corresponding Control value; all other differences are non-significant.

**Table 2:** Individual patient characteristics.

No	Code	Group	Age/Sex	*Ante-mortem* assessment of brain disease/mental state	Cause of death	Braak Stage (Amyloid load)	PMD (h)	Brain Wt (g)
1	H155	Control	61/M	No brain disease or dementia	Ischemic heart disease	0 (0)	7.0	1258
2	H121	Control	64/F	No brain disease or dementia	Pulmonary embolism	0 (0)	5.5	1260
3	H132	Control	63/F	No brain disease or dementia	Ruptured aorta	0 (0)	12.0	1280
4	H122	Control	72/F	No brain disease or dementia	Emphysema	0 (0)	9.0	1230
5	H204	Control	66/M	No brain disease or dementia	Ischemic heart disease	0 (0)	9.0	1461
6	H241	Control	76/F	No brain disease or dementia	Metastatic carcinoma	II (A3, B1, C1)	12.0	1094
7	H164	Control	73/M	No brain disease or dementia	Ischemic heart disease	0 (0)	13.0	1315
8	H123	Control	78/M	No brain disease or dementia	Ruptured aortic aneurysm	0 (0)	7.5	1260
9	H150	Control	78/M	No brain disease or dementia	Ruptured MI	0 (0)	12.0	1416
10	AZ42	AD	60/M	Alzheimer’s dementia	Alzheimer’s disease	VI (3/3)	7.0	1020
11	AZ71	AD	62/F	Alzheimer’s dementia	Alzheimer’s disease	VI (3/3)	6.0	831
12	AZ48	AD	63/F	Alzheimer’s dementia	Bronchopneumonia	VI (2/3)	7.0	1080
13	AZ72	AD	70/F	Alzheimer’s dementia	Lung cancer	V (3/3)	7.0	1044
14	AZ90	AD	73/M	Alzheimer’s dementia	GI hemorrhage	IV (3/3)	4.0	1287
15	AZ96	AD	74/F	Alzheimer’s dementia	Metastatic cancer	V (3/3)	8.5	1062
16	AZ39	AD	74/M	Alzheimer’s dementia	Pseudomonas bacteremia	VI (2/3)	12.0	1355
17	AZ80	AD	77/M	Alzheimer’s dementia	Myocardial infarction	VI (3/3)	4.5	1180
18	AZ38	AD	80/M	Alzheimer’s dementia	Bronchopneumonia/ pulmonary oedema	V (3/3)	5.5	1039

Abbreviations: GI, gastrointestinal; MI, myocardial infarction; PMD, post-mortem delay; wt, weight. Cause of death was determined by post-mortem examination, and brain pathology and Braak Stage were determined by specialist neuropathological examination. All AD cases had ‘Age-Related Plaque’ scores of “C”. Causes of death were the primary causes listed on the death certificate. Patient H241 had *post-mortem* signs consistent with AD and was therefore diagnosed with preclinical disease: the corresponding data have been retained in the main analysis presented in the manuscript, and removed from the control group for the secondary analysis. Control patient H241 (preclinical AD) was described as A3, B1, C1 using the ‘ABC’ criteria for AD neuropathologic change that incorporates histopathological assessments of Aβ deposits (A), staging of neurofibrillary tangles (B), and scoring of neuritic plaques (C)^2^.

**Table 3:** Study of *post-mortem* human brain: relative fold-changes in glucose, sorbitol and fructose in seven brain regions from nine Alzheimer’s cases and nine controls.

Metabolite	Brain-region	Estimate	Lower Bound	Upper Bound	*P*-value	BH FDR *Q*-value
Glucose	Cerebellum	5.2	1.4	18.6	0.015	0.015
	Entorhinal cortex	7.4	2.4	23.2	0.0028	0.0041
	Cingulate gyrus	8.0	2.4	26.1	0.0023	0.0037
	Hippocampus	8.9	2.9	27.4	0.0016	0.0030
	Sensory cortex	9.2	3.0	28.8	0.0014	0.0029
	Motor cortex	11.8	3.7	37.7	0.0006	0.0019
	Middle temporal gyrus	16.4	5.2	51.2	0.0002	0.0019
Sorbitol	Cerebellum	3.7	1.6	8.6	0.0036	0.0044
	Entorhinal cortex	4.3	2.0	9.1	0.0006	0.0019
	Cingulate gyrus	3.1	1.5	6.5	0.0048	0.0053
	Hippocampus	4.1	1.9	8.5	0.0009	0.0021
	Sensory cortex	4.1	1.9	8.6	0.0009	0.0021
	Motor cortex	3.0	1.4	6.6	0.0090	0.0094
	Middle temporal gyrus	3.3	1.5	6.9	0.0039	0.0046
Fructose	Cerebellum	5.3	2.0	14.4	0.0018	0.0031
	Entorhinal cortex	5.7	2.4	13.5	0.0004	0.0019
	Cingulate gyrus	3.9	1.7	9.2	0.0030	0.0041
	Hippocampus	5.5	2.3	12.9	0.0005	0.0019
	Sensory cortex	5.4	2.4	12.4	0.0004	0.0019
	Motor cortex	4.2	1.7	10.2	0.0032	0.0041
	Middle temporal gyrus	5.7	2.6	12.9	0.0003	0.0019

Estimates and their lower and upper bounds were derived by Bayesian modelling. This table incorporates values from n = 9 patients with clinical diagnoses of AD and n = 9 matched asymptomatic controls. Abbreviation: ‘BH FDR *Q*-value’ is the Benjamini-Hochberg False- Discovery Rate-adjusted *P*-value.
