# Supplementary material for: Elevation of brain glucose and polyol-pathway intermediates with accompanying brain-copper deficiency in patients with Alzheimer’s disease: metabolic basis for dementia
Source: Sci Rep. 2016 Jun 9;6:27524. doi: 10.1038/srep27524 (PMC4899713; doi:10.1038/srep27524)
Supplement: Supplementary Information [file srep27524-s1.pdf]

Supplementary Material

Elevation of brain glucose and polyol-pathway intermediates with accompanying brain-copper deficiency in patients with Alzheimer's disease: metabolic basis for dementia

Jingshu Xu,[1,2,3] Paul Begley,[3] Stephanie J. Church,[3] Stefano Patassini,[1,2,3] Selina McHarg,[3] Nina Kureishy[3], Katherine A. Hollywood,[3] Henry J. Waldvogel,[2] Hong Liu,[1] Shaoping Zhang,[1] Wanchang Lin,[3] Karl Herholz,[4] Clinton Turner,[5] Beth J. Synek,[2,5] Maurice A. Curtis,[2] Jack Rivers-Auty,[4] Catherine B. Lawrence,[4] Katherine A. B. Kellett,[4] Nigel M. Hooper,[4] Emma R. L. C. Vardy,[6] Donghai Wu,[7] Richard D. Unwin,[3] Richard L. M. Faull,[2,*] Andrew W. Dowsey,[3*] and Garth J. S. Cooper[1,2,3*#]

S1 Supplementary methods

S1.1 Sample preparation for metabolomic analysis. Aliquots of 50±5 mg wet brain tissue in "Safe-Lok" microfuge tubes (Eppendorf AG; Hamburg, Germany) underwent a Folch-style extraction using a TissueLyser (Qiagen; Manchester, UK). Each sample was extracted in 0.8 mL 50:50 (v/v) methanol:chloroform to which a solution of the labelled internal standards in methanol had been added to 0.016 mg/mL (final w/v) of each internal standard in the extraction solvent (10 min, 25 Hz) with a single 3-mm tungsten carbide bead. Samples from each brain region were handled as single, separate batches for this and all subsequent procedures. Separation of phases was by addition of 0.4 mL water followed by vortex-mixing (10-15 s) and centrifugation (2,400 g, 15 min). After separation, tissue debris lay at the interface between the lower (non-polar, chloroform) and upper (polar, methanol:water) phases. Extraction blanks were prepared by including microfuge tubes without tissue samples within each batch. The chloroform supernatant was removed and stored for LC-MS analysis (to be reported elsewhere).

Residual chloroform was removed using a 500 µL HPLC syringe (Sigma Aldrich). Tubes were centrifuged (16,000 g, 15 min) to pellet tissue debris. From the methanol:water supernatant, 200-µL aliquots were transferred to pre-labelled tubes containing a further 600-µL methanol to precipitate residual protein. A QC pool was constructed from further 200-µL aliquots from each extraction, gently mixed, and 200 µL dispensed into tubes containing 600-µL methanol. After brief mixing, both sample and QC tubes were centrifuged (16,000 g, 15 min). 750-µL aliquots of each were then transferred to a final set of pre-labeled tubes, and dried (~30°C, 16-18 h) in a centrifugal concentrator (Savant Speedvac; Thermo Scientific). Dried residues were then held in sealed tubes at 4°C until derivatization for GC-MS analysis.

S1.2 GC-MS analysis of tissue metabolites. Methyloxime/trimethylsilyl derivatives were prepared by a two-step procedure [1]. GC-MS analysis was performed using an MPS2 autosampler (Gerstel; Mülheim an der Ruhr, Germany), a Gas Chromatograph with Split/Splitless inlet (Agilent 7890A; Santa Clara, CA), and a time-of-flight mass spectrometer (LECO Pegasus HT; Stockport, UK) by a method that we previously described [2], optimized for the separation of glucose, sorbitol and fructose by adjustment of chromatographic conditions and validation by use of standard reference materials to ensure adequate separation of these analytes from one another.

Gas chromatography was performed using a J&W DB-17MS column (30 m x 0.25 mm x 0.25 µm; Agilent: #122-4732) with a 3-m deactivated Fused-Silica retention gap (0.25 mm; Agilent: #160-2256-10), and helium carrier gas (1.4 mL/min, constant-flow mode). Prior to sample analysis, the GC-MS was prepared for use as described [1]. 1-µL sample injections were made in Pulse Splitless mode at an inlet temperature of 270°C, using an "empty, hot-needle" technique. Initial column temperature was 50°C, held for 6 min and then ramped to 300°C at 10°C/min and held for a further 4 min; this resulted in a total cycle time of 42 min between injections. After an initial 450-s solvent delay (to allow solvent and reagents to elute without damaging the detector), mass spectra were acquired (rate of acquisition, 10 spectra/s; mass range, 45-800 Da; energy, 70 eV; temperature, 220°C).

Measurements were made in a series of single-batch experiments, wherein each brain region constituted a batch. Within each batch, individual cases and controls were randomized, and run in a sequence interleaved with injections of pooled QC samples (one per four study samples) and extraction blanks (two/batch), and a conditioning lead-in sequence of six QC injections at the start of each batch. Extraction blanks were inspected visually to confirm absence of carryover, but not included in subsequent data analysis.

S1.3 Data reduction for GC-MS. Data were prepared using the 'Reference Compare' method within ChromaTOF 4.5 (LECO). Briefly, the software was used to perform a global peak deconvolution of representative samples based on pre-defined parameters to compile a list of nominated 'metabolites', and search mass-spectral libraries to generate putative identities. Data bases employed were: the NIST Mass Spectral Reference Library (NIST08/2008; National Institute of Standards and Technology/Environmental Protection Agency/National Institutes of Health Spectral Library; NIST, Gaithersburg, MD, USA); the Golm Metabolome Database (Max Planck Institute of Molecular Plant Physiology, Potsdam-Golm, Germany); and an in-house library [1]. Chromatographic retention-time data were available from reference standard compounds for a subset of the identities, and matching of both mass spectra and expected retention time(s) was interpreted to constitute a definitive molecular identification. For glucose, sorbitol and fructose, we performed definitive identification by comparison with authentic synthetic standards (G5400, 240850, and F0127 respectively; Sigma Aldrich).

From the list of nominations, we compiled reference tables comprising expected mass spectra and retention-time windows describing 69 confidently-identified metabolites; these were then applied as target lists of features to be searched across all the samples. As pooled QC samples should contain all metabolite features encountered in the experiment, these are suitable for compiling reference tables. To provide a robust reference table, the initial list of nominations was edited to remove ambiguous and low-quality spectra prior to application. Global deconvolution was performed on several (3-4) pooled QC injections across the entire experiment to improve molecular identifications.

By displaying these overlaid while editing the list, reproducible spectra were thus more readily distinguished from lower-quality candidates. The same target list was used for all brain regions.

S1.4 Determination of metabolite abundance In order to use the edited reference table as an analytical tool, appropriate parameters such as mass-spectral match-thresholds and tolerable retention-time deviations were specified, and the table initialized using a pooled-QC sample to provide reference m/z peak areas. Improved reproducibility was achieved by use of internal-standard ratios rather than raw peak-areas. Here, a set of seven isotopically-labeled standards (citric acid-d4, [^{13}C]-6-D-fructose, L-tryptophan-d5, L-alanine-d7, stearic acid-d35, benzoic acid-d5, and leucine-d10), purchased from Cambridge Isotopes Inc (Tewksbury, MA), were used. By determining which internal standard gave the lowest variance for a given metabolite across all the QC injections, the most suitable standard was assigned to each metabolite.

Data for each experiment were compiled into a matrix of metabolite-intensity data, which were merged with experimental metadata for visualization and statistical analysis. Although the automated procedure is highly reliable (estimated return of correct peak areas for >95% of features measured), data sets were also curated manually to remove possible integration errors. Initial data analysis was conducted using a set of in-house procedures running in the R environment and a Principal-components Analysis (PCA) visualization was used to confirm overall data integrity. Multiple-comparison analyses were performed to compare metabolite intensities between classes, applying a false discovery rate (FDR) correction.

S 1.5 Sample preparation for metal content analysis. Brain samples of 50 ± 5 mg wet-weight were dried to constant weight in a centrifugal concentrator (Savant Speedvac; Thermo-Fisher, Waltham, MA). Dried samples were digested using an 'open vessel' method. All sample containing tubes and two blank tubes were punctured on the lid to avoid pressure build-up during the procedure. To each tube, 0.2 mL of Trace Metal Grade concentrated nitric acid (A509 Trace Metal Grade; Fisher, Loughborough, UK) containing (5% v/v) Agilent Internal Standard mixture (5183-4681; Agilent Technologies, Cheadle, UK) was added. Tubes were then inserted into a "Dri-block" heater at room

temperature, set to 60°C for the first 30 min and 100°C for a further 210 min. After digestion, the tubes were allowed to cool to room temperature and 100 µL-aliquots taken from each digestion solution and added to 15 mL Falcon tubes (525-0629, VWR Lutterworth, UK) containing 5 mL LC-MS grade water, to produce solutions for analysis at a final nitric acid concentration of 2% (v/v).

S 1.6 ICP-MS analysis of tissue-copper level. Tissue metal concentrations were measured using an Agilent 7700x ICP-MS spectrometer equipped with a MicroMist nebulizer (Glass Expansion, Melbourne, Australia) and a Scott double pass spray chamber. Nickel sample and skimmer cones were used. Sample introduction was performed using an Agilent Integrated autosampler (I-AS). A multi-element method including all elements present in the calibration solution, as previously reported by our group [3], was applied. Calibration solutions were produced by appropriate dilutions of Environmental Calibration Standard (Agilent 5183-4688). Helium (He) was used as collision gas; copper levels were analyzed in He-mode (5.0 mL min^{-1} helium) with an integration time of 0.3 s. For each analytical batch, multi-element calibration was performed using serial dilutions of the calibration standard. An intermediate concentration from this calibration series was used as a periodic quality control (QC) sample throughout each analytical batch. Instrument and digestion blanks were also interspersed through each set of randomized samples.

S1.7 Statistical methods for brain metabolite study. Two chromatographic peaks were observed for each of glucose and fructose, due to the formation of isomeric forms during derivatization. For each, the peak least susceptible to mass spectral interference was selected as a measure of the metabolite's abundance. In the selected glucose, sorbitol and fructose signals, a small number of peaks with integration errors were corrected by manually selecting the correct peak for subsequent quantification.

Post-mortem human and rat data were both log-transformed, and normality and outliers evaluated by QQ plots and Cook's D-statistic respectively. Initial assessment of differential expression of metabolites between case and control samples was performed with Welch's unequal-variance *t*-test on each metabolite from each whole rat brain and each *post-mortem* human brain region separately. This approach showed that metabolite levels were elevated in the case group in every comparison (two-

sided test, 5% significance level, with false discovery rate adjustment by the Benjamini-Hochberg method). Nevertheless, for the human samples, QQ plots indicated a clear intra-subject correlation across brain regions. We therefore modeled this effect by constructing a linear mixed-effects model (PROC MIXED, SAS 9.3, SAS Institute, Cary, NC) for each of glucose, sorbitol and fructose. Model building was guided by using the Akaike Information Criterion corrected for finite sample size (AICc). For each metabolite, a subject random effect was added. In addition, for glucose the subject effect was markedly stronger in the case group, so that a separate random effect for cases and controls was found to be suitable. With subject effect(s) fitted, AICc indicated that separate residual variances for each brain region were warranted but separate residual variances for cases and controls within each region were not. Model fitting was performed with Restricted Maximum Likelihood (REML), while fixed effect significance testing was conducted using Wald's *t*-statistic with Satterthwaite approximation and Kenward-Roger adjustment, followed by Benjamini-Hochberg FDR correction. For the case-control comparisons (Table 3, Suppl. Table S1), brain region main effect and condition-region interaction were treated as fixed. For comparison between brain regions (Fig. 2), an additional condition main effect was added.

To further understand variation in relative fold-change both within and between brain regions, posterior probability density curves were generated (Fig. 2), by sampling the models specified above with Bayesian Markov-Chain Monte Carlo approach [4]. Residual variances were assigned inverse-Gamma priors, while random effects were assigned parameter-expanded Cauchy priors. The model was tested with different prior scale factors to establish that the weak priors were not informative to the outcome. Thorough mixing was assured by generating 300,000 warmup samples followed by 1,000,000 inferential samples.

S1.8 Statistical methods employed for the case-control study. This analysis was performed using IBM SPSS v22. Distributions of variables were tested for skewness using the Kolmogorov-Smirnov Goodness-Of-Fit test, for 42 AD subjects and 43 matched controls (Suppl. Table S2). Parametric data are presented as mean±standard error of the mean (SEM), and non-parametric data as median (range). Comparisons between AD and control groups were performed using an Independent-Samples *t*-test

for parametric data and the Mann-Whitney U test for non-parametric data. Pearson's X^2-Squared analysis was performed on categorical data.

S 1.9 Statistical methods for brain copper content analysis. The dataset was exported to excel worksheets and individual values of each sample were normalized by the corresponding sample weight (dry weight as determined after the drying procedure). Weight-adjusted datasets were then log-transformed for statistical analysis. Means (±95% CI) of the log-transformed data were calculated and the significance of between-group difference was determined by unpaired *t*-test with Welch's correction to allow for unequal variance and sample sizes. Means (±95% CI) were back-transformed to reflect the actual concentrations of elements. Statistical calculations were performed using GraphPad v6.04 (Prism; La Jolla, CA).

S2. Supplementary results

S2.1 Metabolite levels in human brain. We categorized 69 metabolite features with definitive or confident identifications [1]. PCA revealed excellent class separation in all brain regions between cases and controls in AD and experimental diabetes (results not shown). Of the identified metabolites, 55 were shown to change (FDR-corrected multiple *t*-test, $P<0.05$) in one or more AD brain regions: of these, between 16 and 33 metabolites were identified as significantly changed in any brain region. Perhaps surprisingly, changes in metabolite levels of cerebellum (relative spared in AD) were similar to those in other regions, consistent with a pervasive metabolic defect in AD. A full consideration of all metabolite changes observed in this work is currently being prepared and is beyond the scope of this report: here we have focused on glucose, fructose and sorbitol in light of the known epidemiological links between altered glucose metabolism, AD and dementia [5-7].

S2.2 Metabolite levels in rat brain. Mean (±95% CI) fold-elevations in diabetic rat-brain compared to control values were: glucose, 5.3 (3.1-9.1; $P<0.0001$ *vs* control); sorbitol, 5.8 (3.5-9.5; $P<0.0001$); and fructose, 4.6 (3.0-7.0; $P<0.0001$) (Fig. 2b, Suppl. Table S1). Levels of glucose, sorbitol and fructose were all markedly elevated in whole-brain extracts from diabetic rats, consistent with the presence of a global defect in regulation of the polyol pathway in AD brain. Moreover, levels of these

metabolites in diabetic and control rat-brain tissue showed patterns closely resembling those in human AD brain and respective controls. The elevations in glucose, sorbitol and fructose in human AD mirrored those in diabetic rat brain and other tissues from diabetic patients [8,9]. These close similarities point to common metabolic mechanisms causing tissue damage and dementia in patients with AD or T2D. Tissue acquisition was tightly controlled in these diabetic rats, so the findings provide support for the measurements in human brain, and indicate that *post-mortem* change is unlikely to provide an explanation for the observed elevations in polyol-pathway intermediates in AD cases.

***S3* Supplementary Figure Legend**

Supplementary Figure S1 We employed a systematic approach for microdissection of human-brain as shown here, to ensure between-brain consistency in acquisition of analytical samples from different subjects. Briefly, for each case, seven blocks containing the regions of interest were carefully selected one-by-one from pre-dissected brain tissue available in the University of Auckland Brain Bank. Particular attention was paid to ensure matching of anatomical level in each block for each region across all 18 brains. Subsequently, each region was systematically dissected, as shown in **A-G**. In total, seven samples, each of 50 (± 5) mg wet-weight, were generated from each brain region for each subject. Samples labeled 1 (*green*) were analyzed for metabolite content by GC-MS in wet tissue, whereas those numbered 6 (*pink*) were analyzed by ICP-MS for metal content in dry tissue. We took great care to sample grey matter only and to minimize potential contamination with white matter; however, we cannot exclude small amounts of white-matter-derived material in cerebellar samples, as precise separation of grey and white matter therein is not easily achievable owing to the complex anatomy.

S4 Supplementary Tables

S4.1 Supplementary Table S1

Suppl. Table S1

Results of Bayesian analysis showing mean fold-increases and corresponding upper and lower bounds of glucose, sorbitol and fructose in whole-brain extracts from diabetic rats compared with nondiabetic-control rats

Metabolite	Mean	Lower Bound	Upper Bound	*P-value*
Glucose	5.3	3.1	9.1	0.0001
Sorbitol	5.8	3.5	9.5	0.0001
Fructose	4.6	3.0	7.0	0.0001

Data are from diabetic rats (n=7) and nondiabetic controls (n=7)

***S4.2* Supplementary Table S2**

Suppl. Table S2

Characteristics of participants in a case-control study of AD

Characteristic	AD	Control	*P-value*
Number	42	43	-
Male (%)	52.4	53.5	NS
Age (y)	78.2±1.3	78.1±1.1	NS
MMSE score	21 (11-25)	29 (27-30)	<0.001
ApoE4 allele-positive (%)	71.4	32.6	<0.001
Fasting plasma glucose (mmol/l)	5.0 (3.8-10.6)	5.1 (4.2-8.9)	NS
Serum HbA1c*	5.6 (5.1-7.8)	5.7 (5.1-8.4)	NS
Fasting plasma copper (µmol/l)*	13.8 (12.9-14.6)	14.7 (13.6-15.8)	NS

Data are mean±SEM, median (range) or mean (±95% CI). * n = 35 in AD group. Nonstandard abbreviations: MMSE, Mini-Mental State Examination; NS, not significant

_S5_ Supplementary Figure S1

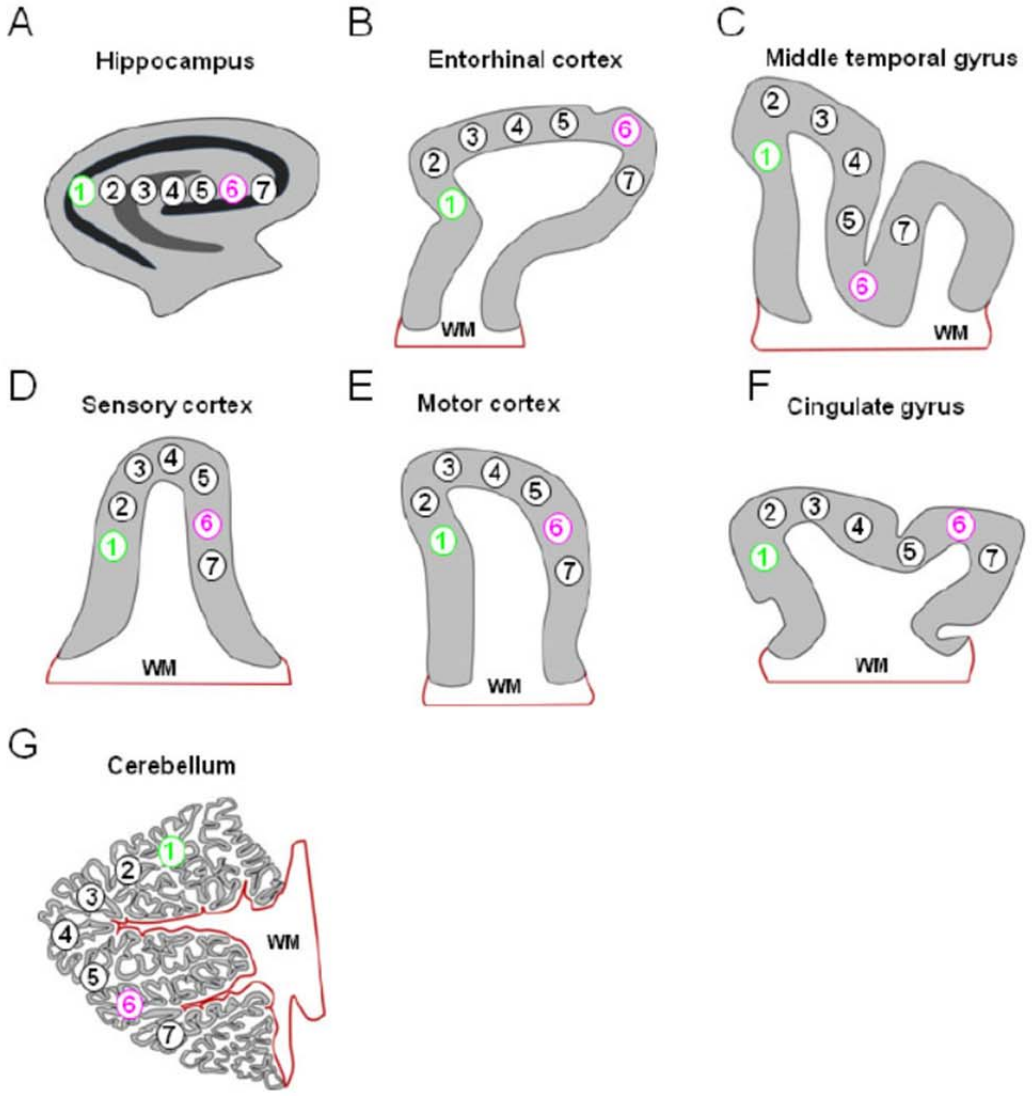

***S6* Supplementary References**

1 Dunn, W. B. *et al.* Procedures for large-scale metabolic profiling of serum and plasma using gas chromatography and liquid chromatography coupled to mass spectrometry. *Nature Protocols* **6**, 1060-1083 (2011).

2 Begley, P. *et al.* Development and performance of a gas chromatography-time-of-flight mass spectrometry analysis for large-scale nontargeted metabolomic studies of human serum. *Anal Chem* **81**, 7038-7046 (2009).

3 Church, S. J. *et al.* Deficient copper concentrations in dried-defatted hepatic tissue from ob/ob mice: A potential model for study of defective copper regulation in metabolic liver disease. *Biochem Biophys Res Commun* **460**, 549-554, (2015).

4 Hadfield, J. D. MCMC Methods for Multi-Response Generalized Linear Mixed Models: The MCMCglmm R Package. *J Stat Software* **33**, 1-22 (2010).

5 Hoyer, S. & Nitsch, R. Cerebral excess release of neurotransmitter amino acids subsequent to reduced cerebral glucose metabolism in early-onset dementia of Alzheimer type. *J Neural Transm* **75**, 227-232 (1989).

6 Ott, A., Breteler, M. M., van Harskamp, F., Stijnen, T. & Hofman, A. Incidence and risk of dementia. The Rotterdam Study. *Am J Epidemiol* **147**, 574-580 (1998).

7 Huang, C.-C. *et al.* Diabetes mellitus and the risk of Alzheimer's disease: a nationwide population-based study. *PLOS ONE* **9**, e87095 (2014).

8 Gabbay, K. H. Hyperglycemia, polyol metabolism, and complications of diabetes mellitus. *Annu Rev Med* **26**, 521-536 (1975).

9 Brownlee, M. The pathobiology of diabetic complications: a unifying mechanism. *Diabetes* **54**, 1615-1625 (2005).
